# Women's Social Well-Being During Pregnancy: Adverse Childhood Experiences and Recent Life Events

**DOI:** 10.1089/whr.2022.0023

**Published:** 2022-06-13

**Authors:** Cheryl Buehler, Savannah A. Girod, Esther M. Leerkes, Lauren Bailes, Lenka H. Shriver, Laurie Wideman

**Affiliations:** ^1^ Department of Human Development and Family Studies, University of North Carolina Greensboro, Greensboro, North Carolina, USA.; ^2^ School of Health and Human Sciences, University of North Carolina Greensboro, Greensboro, North Carolina, USA.; ^3^ Department of Psychological Sciences, Vanderbilt University, Nashville, Tennessee, USA.; ^4^ Department of Nutrition, University of North Carolina Greensboro, Greensboro, North Carolina, USA.; ^5^ Department of Kinesiology, University of North Carolina Greensboro, Greensboro, North Carolina, USA.

**Keywords:** pregnant, social, well-being, health, ACEs, life events

## Abstract

**Background:**

Adverse experiences during childhood and recent stressful life events are each associated with women's reduced well-being and poorer health during pregnancy. Few studies, however, have focused upon pregnant women's social well-being, and inclusion of both independent variables in the same analysis is rare. This study focuses upon adverse experiences during childhood as well as recent life events in relationship to four aspects of social well-being: social support, couple aggression for partnered women, neighborhood safety, and food insecurity.

**Materials and Methods:**

A diverse community sample of 176 pregnant women completed questionnaires during their third trimester. A cross-sectional design was used that included retrospective reports of childhood experiences, as well as reports of recent life events and current well-being.

**Results:**

Adverse experiences during childhood were uniquely associated with couple aggression (β = 0.206, *p* = 0.026) and lower neighborhood safety (β = −0.185, *p* = 0.021). Recent stressful life events were uniquely associated with lower social support (β = −0.247, *p* = 0.001) and greater food insecurity (β = 0.494, *p* = 0.000). For social support and food insecurity, there was a significant indirect pathway from adverse childhood experiences through recent stressful life events. Adverse child experiences and recent stressful life events did not interact.

**Conclusions:**

A life-course perspective that considers women's experiences across their life span is critical for use by both researchers and health practitioners. Adverse childhood experiences and recent stressful life events are important for understanding social features of pregnant women's daily lives.

## Introduction

Women's health and well-being during pregnancy are central areas of interest and concern in the United States and internationally.^1,2^ The social involvements and environments that constitute pregnant women's everyday lived experiences shape their well-being.^3,4^ As such, understanding a pregnant woman's health and well-being is fostered by explicating unique features of their social experiences.^5,6^ In the current study, we address four salient aspects of women's everyday social experiences: social support, couple aggression for partnered women, neighborhood safety, and household food insecurity. These four aspects highlight women's social experiences at various levels of their personal and relational ecologies and have important implications for women's antepartum physical and mental health, as well as infant's well-being.^3,5–7^

Two central correlates of women's social well-being include adverse experiences during childhood and stressful life events during pregnancy. Adverse childhood experiences place women, regardless of pregnancy status, at greater risk for negative social experiences.^8,9^ Pregnancy is a particularly impactful developmental period for adverse childhood experiences because women's health and well-being are critical for both their own and their infants' healthy functioning.^10–12^ Sparse literature has focused upon the social aspects of women's lives, but a recent analysis suggests adverse experiences during childhood are associated with less positive close relationships, greater relationship aggression, and inadequate economic resources.^13^ Specifically, childhood adversity has been associated with pregnant women's romantic relationship conflict (controlling for poverty), greater psychosocial difficulties (a multidimensional index that included lower social support), and lower income.^7,14^

However, food insecurity, as a proximal feature of socioeconomic disadvantage, has not been examined in this literature. Addressing food security as a marker of socioeconomic circumstances is important because it has integral connections with women's and fetal health and well-being.^15,16^ In addition, perceived neighborhood safety might be important,^17^ in part, because perceived safety may facilitate physical activity and enhance social connections.^18,19^ The current study addresses existing gaps in the understanding of childhood adversity and pregnant women's social well-being by examining social support, couple aggression, neighborhood safety, and household food insecurity.

In addition to considering childhood adversity, we also examine the association between stressful life events experienced during pregnancy and women's social well-being. Data from the Pregnancy Risk Assessment Monitoring System suggest that 65%–70% of women experienced at least one stressful life event during pregnancy and about 20% were classified into a multiple stressor group.^20^ Studies focused on women with substance-related health problems report that pregnant women experienced an average of two to five stressful life events during the past year.^21,22^ Reduced global well-being in pregnant women has been associated with stressful life events experienced during the previous year^23,24^ and compromised infant development.^25^

In the scant research examining pregnant women's social well-being, experiencing cumulative stressful life events correlated with lower social support,^24^ physical abuse from partner,^26^ and lower neighborhood quality.^17^ Salas-Wright et al. highlighted the need for research that better addresses social factors (including socioeconomic disadvantage) that contribute to women's vulnerabilities during pregnancy.^27^ The current study heeds this call by examining the infrequently studied social features of social support, couple aggression, neighborhood safety, and household food insecurity.

Although accumulating evidence suggests that adverse childhood experiences and stressful life events during pregnancy each negatively affect women's health and well-being, limited research has considered these risk factors conjointly in the same analysis. As such, little is known regarding how these two risk factors interconnect or their potential unique impacts on women's well-being during pregnancy. Fewer than 10 studies published during the past decade examined pregnant women's well-being in relationship to adverse childhood experiences and recent stressful life events. This scant literature suggests that examining both risk factors in the same model is illuminating. For example, adverse childhood experiences were uniquely associated with pregnant women's cortisol awakening response (controlling for recent stressors), but not with the diurnal cortisol pattern across the day.^28^

Using a composite measure of psychosocial risk that included anxiety, perceived stress, mental health difficulties, depression, and lower social support, Racine et al. found that adverse childhood experiences were associated with mother's self-reported hostile behavior toward their infant indirectly through pregnancy psychosocial risk.^29^ Other markers of well-being examined in relationship to both adverse childhood experiences and recent life events include pregnant women's inflammation, dysregulated emotional well-being, and preterm birth.^30–32^ Taken together, this evidence suggests that when considered conjointly, adverse childhood experiences and concurrent stressors have implications for maternal and fetal health during pregnancy and postbirth. Additional research is warranted to elucidate how adverse childhood experiences and recent life events are conjointly related to pregnant women's social well-being.

**FIG. 1.**
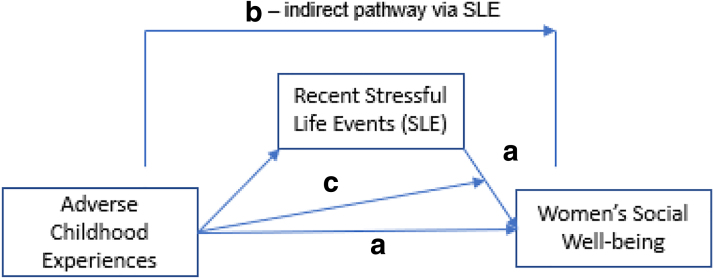
Study aims focused upon pregnant women's social well-being, adverse childhood experiences, and stressful life events during pregnancy. **(a)** Aim 1; **(b)** Aim 2; **(c)** Aim 3.

Given the importance of women's social well-being during pregnancy and the dearth of information regarding the relative roles of adverse childhood experience and recent stressful life events, this study examines three research aims. One aim is to examine the unique associations with two risk factors: adverse childhood experiences and recent stressful life events (Fig. 1a). We hypothesize that adverse childhood experiences and their recent stressful life events are each uniquely associated with pregnant women's social well-being. A second aim is to examine whether stressful life events during pregnancy help explain the association between adverse childhood experiences and a given marker of women's social well-being (*i.e.*, indirect effect; Fig. 1b). We hypothesize that the indirect pathway from adverse childhood experiences to women's social well-being through stressful life events is statistically significant.

A third aim is to examine whether adverse child experiences function to exacerbate the hypothesized deleterious association between recent stressful life events and pregnant women's social well-being (*i.e.*, interactive effect; Fig. 1c). We hypothesize that the association between recent stressful life events and a particular aspect of women's social well-being is exacerbated by adverse experiences during childhood.

## Materials and Methods

### Study design and data

The current study used survey data from a community-based sample of 176 pregnant women living in the southeastern United States. Pregnant women were recruited during their third trimester from (a) childbirth education classes at local hospitals and the Public Health Department, (b) prenatal breastfeeding classes provided by the Special Supplemental Nutrition Program for Women, Infants, and Children, (c) notices in waiting rooms of obstetrics and gynecology practices and stores/events targeting expectant parents, (d) social media, and (e) paid advertising in regional print and online media focused on pregnancy and parenting. Eligibility criteria were (a) 18 years of age or older, (b) expecting a singleton, (c) written English comprehension, and (d) plans to stay in the region for at least 3 years.

Women ranged in age from 18 to 49 years (*M* = 29.16, SD = 6.05). Educational attainment ranged from some high school through graduate schooling; 22% of the women did not have formal schooling past high school; and the average level of education was some college. The sample had a wide range of annual income with a median household income of $45,000. About 30% of the women lived in poverty (using an income-to-needs indicator).

With regard to race, 46% self-identified as White, 32% self-identified as Black/African American, and about 22% self-identified as another race, including multiracial. With regard to marital status, 52% of the women were married and an additional 24% cohabited with their romantic partner. About 14% were single and currently not in a romantic relationship. The remaining women were separated/divorced (<2%) or in a serious romantic relationship but not cohabiting (8%). This sample was comparable to women in the county with regard to race and Hispanic ethnicity, although they had more formal education but lower incomes.^33^

Upon enrollment, data were collected during the last couple of months of pregnancy using a cross-sectional data collection protocol. Online Qualtrics questionnaires assessed sociodemographic characteristics, adverse childhood experiences, stressful life events during pregnancy, and current social well-being. These questionnaires were completed before laboratory visits. The study was approved by the university Institutional Review Board (protocol #18-0198) and additional detail can be found in the overall study.^34^

### Measures

The four measures of pregnant women's social well-being were the primary outcomes of interest. Current social support was assessed using the 10-item short form of the Social Provisions Scale.^35^ The response format ranged from 1 (*strongly disagree*) to 4 (*strongly agree*), and a higher score indicated greater social support (α = 0.97). Couple aggression was assessed for partnered women using a 12-item measure that captured both self and partner hostile behaviors such as yelling and threatening.^36^ The response format ranged from 1 (*never*) to 5 (*always*), and a higher score indicated greater expressed hostility and aggression (α = 0.89).

Current perceptions of neighborhood safety was assessed using the three-item safety subscale of the Neighborhood Conditions/Qualities Scale.^37^ The response format ranged from 1 (*strongly disagree*) to 5 (*strongly agree*), and a higher score indicated greater perceived safety in the neighborhood (α = 0.87). The summary scores for these three social well-being variables were created using mean scores to retain the original response scale metric. Food insecurity during pregnancy was assessed using the six-item Short Form Food Security Survey.^38^ The rescaled response format ranged from 0 to 1, and a higher score indicated greater food insecurity (α = 0.87).

Adverse childhood experiences were assessed using the 10-item adverse childhood experiences measure from the Centers for Disease Control and Prevention, 2016.^39^ Women were asked to think about their first 18 years of life when reporting experiences related to maltreatment, abuse, and challenging household conditions such as caregiver substance problems, incarceration, or mental health problems. Positive endorsements were summed, and a higher score indicated greater adverse experiences during childhood. Retrospectively reported adverse childhood experiences corresponded moderately with prospectively reported experiences.^8^ This measure was an index and so accumulation rather than interitem consistency was a central psychometric attribute.

Recent stressful life events were assessed using the 26-item Stressful Life Events Questionnaire developed for use with pregnant women.^25^ Sample events included having a serious accident or illness, trouble with the law, becoming homeless, and death of a family member or close friend. Positive endorsements were summed with higher scores indicating greater accumulation of life events since becoming pregnant. When couple aggression was the dependent variable for partnered women, four items focused on relationship hostility and aggression were removed from the life events summary score so as not to confound the two measures.

### Analytic procedures

Preliminary analyses considered five potential covariates that might be associated with pregnant women's social well-being: women's age, educational attainment, primiparous status, household size, and self-identified race-ethnicity.^40,41^ These five potential controls correlated with each of the four measures of women's social well-being. Our decision rule was that a covariate was included in all further regression analyses if it was correlated (*p* < 0.05) with any one of the four social well-being measures. This process eliminated household size from additional analyses.

Each women's social well-being variable was examined in a separate regression and/or path analysis using Mplus.^42^ The unique main effects of adverse childhood experiences and recent stressful life events (Fig. 1) were estimated using multiple regression to address the first aim. The indirect pathway from adverse childhood experiences to pregnant women's social well-being was estimated using path analysis. The indirect effect was estimated using Sobel's formula, and the confidence interval for the unstandardized coefficient was estimated using bootstrapping, with 1,000 draws.^43^ The moderating effect of adverse childhood experiences was estimated by including an interaction term (created using grand-mean centered variables) in the path analysis. The small amount of missing data (0%–6% across study variables) was handled using full information likelihood estimation.^44^ Models for social support, neighborhood safety, and food insecurity used the full analytic sample (*N* = 176). Only partnered women were included in the model for couple aggression (*n* = 146).

Finally, supplementary analyses were conducted using income-to-needs as the measure of economic disadvantage to provide additional information that can be integrated with previous research.

## Results

Correlations were in the expected directions (Table 1). The correlations among the four aspects of women's social well-being were statistically significant and small-to-moderate in strength. Women's adverse childhood experiences were moderately associated with women's recent stressful life events. Adverse childhood experiences and recent stressful life events were significantly correlated with each aspect of social well-being and were small-to-large in strength.

**Table 1.** Study Variable Means, Standard Deviations, and Correlations

	1	2	3	4	5	6	7	8	9	10
1. Social support	—									
2. Couple aggression^a^	−0.26^**^	—								
3. Neighborhood safety	0.25^**^	−0.23^*^	—							
4. Food insecurity	−0.32^**^	0.29^**^	−0.33^**^	—						
5. Adverse CH experiences	−0.15^*^	0.28^**^	−0.28^*^	0.35^**^	—					
6. Stressful life events	−0.28^**^	0.38^**^	−0.21^**^	0.57^**^	0.45^**^	—				
7. Age	0.10	−0.14	0.18^*^	−0.22^**^	−0.13	−0.25^**^	—			
8. Education	0.13	−0.19^*^	0.36^**^	−0.28^**^	−0.25^**^	−0.23^**^	0.48^**^	—		
9. Primipara status^b^	−0.11	0.16	−0.18^*^	0.09	0.07	−0.02	0.29^**^	−0.18^*^	—	
10. Race^c^	−0.15	0.20	−0.33^**^	0.22^**^	0.13	0.23^**^	−0.28^**^	−0.43^**^	0.24^**^	—
Mean or %^d^	3.47	1.53	3.69	0.20	1.73	2.39	29.15	4.24	55^d^	52^d^
Standard deviation	0.64	0.51	0.88	0.30	2.01	2.38	6.06	1.88	0.50	0.50

^*^ *p* < 0.05; ^**^p < 0.01.

^a^ Partnered women only (*n* = 146).

^b^ 0 = First-time birth; 1 = not a first-time birth.

^c^ 0 = Self-identified white, non-Hispanic; 1 = other self-identified race.

^d^ Percentage for participants scored a “1.”

CH, childhood.

### Aim 1: unique effects of women's adverse childhood experiences and recent stressful life events

For partnered women, adverse experiences during childhood were uniquely associated with greater couple aggression but stressful life events during pregnancy were not associated uniquely with couple aggression (Table 2). This same pattern of unique effects of adverse child experiences was found for perceptions of neighborhood safety (*i.e.*, adverse experiences during childhood were associated with lower perceived safety).

**Table 2.** Adverse Childhood Experiences and Recent Stressful Life Events Predicting Pregnant Women's Social Well-being

Unique effects	Social support^a^			Couple aggression^b^			Neighborhood safety^a^			Food insecurity^a^		
	B	b	*p*	β	b	*p*	β	b	*p*	β	b	*p*
ACEs	−0.016	−0.005	0.830	**0.206**	**0.056**	**0.026**	−**0.185**	−**0.081**	**0.021**	0.084	0.013	0.231
Recent SLE	−**0.247**	−**0.066**	**0.001**	0.103	0.029	0.301	−0.022	−0.008	0.793	**0.494**	**0.063**	**0.000**
Age	0.055	0.006	0.600	−0.122	−0.010	0.260	0.046	0.007	0.606	−0.065	−0.003	0.414
Education	0.008	0.003	0.931	0.026	0.007	0.811	**0.185**	**0.087**	**0.037**	−0.085	−0.014	0.284
Primipara status	−0.121	−0.156	0.140	0.165	0.169	0.078	−0.105	−0.187	0.184	0.096	0.058	0.179
Race-ethnicity	−0.042	−0.054	0.616	0.079	0.081	0.402	−**0.184**	−**0.326**	**0.019**	0.023	0.014	0.745
*R*^2^, %	9.7			12.9			21			36		

Boldface font is used to highlight statistical significance.

^a^ *N* = 176.

^b^ *n* = 146 partnered women only.

ACE, adverse childhood experience; SLE, stressful life events.

The unique main effects showed a different pattern for women's social support and household food insecurity. Stressful life events were uniquely associated with lower social support; however, adverse experiences during childhood were not associated uniquely with women's social support. Women who had experienced more stressful life events during their pregnancy also reported greater household food insecurity. Food insecurity was not uniquely associated with adverse experiences during childhood. These two measures of women's life experiences across the life course, plus the four covariates, explained moderate levels of variance in pregnant women's social well-being (*R*^2^s ranged from 9.7% to 36%).

### Aim 2: the indirect pathway

The indirect pathway from adverse childhood experiences to women's social well-being *via* recent stressful life events was supported for social support (*b* = −0.036, *p* = 0.044, β = −0.112, 95% CI [−0.065 to −0.014]) and food insecurity (*b* = 0.033, *p* = 0.000, β = 0.222, 95% CI [0.020 to 0.052]). As such, stressful life events during pregnancy helped to explain the association between adverse experiences as a child and women's social support and their current household food insecurity.

The indirect effects were not statistically significant for couple aggression (*b* = 0.011, *p* = 0.360, β = 0.042, 95% CI [−0.010 to 0.037]) or neighborhood safety (*b* = −0.006, *p* = 0.663, β = −0.015, 95% CI [−0.041 to 0.021]). Indirect effects were not indicated because there was not a unique statistically significant association between stressful life events during pregnancy and women's reports of couple aggression or perceptions of safety in their neighborhood.

### Aim 3: the interaction between adverse childhood experiences and recent stressful life events

Examining each aspect of women's social well-being in separate analytic models, none of the interaction terms was statistically significant. Across the models, the unstandardized regression estimates for the interaction term ranged from −0.038 (*p* = 0.717; couple aggression) to 0.127 (*p* = 0.121; food insecurity).

### Supplementary analyses

Given previous research has examined income as a marker of socioeconomic disadvantage, we conducted additional analyses that utilized an income-to-needs measure of economic well-being (higher scores indicated greater well-being). The zero-order correlation between food insecurity and income-to-needs in this sample of pregnant women was −0.36 (*p* < 0.001). The pattern of findings from this supplementary regression analysis using an income-to-needs measure partially replicated the food insecurity findings, although the associations were not as strong as for food insecurity.

The unique association with adverse childhood experiences was not statistically significant for income-to-needs (*b* = −0.043, *p* = 0.678, β = −0.029), like the finding for food insecurity (Table 2, *b* = 0.013, *p* = 0.231, β = 0.084). The unique association with stressful life events during pregnancy, however, was not statistically significant for income-to-needs (*b* = −0.149, *p* = 0.095, β = −0.029), but was statistically significant for food insecurity (Table 2, *b* = 0.663, *p* = 0.000, β = 0.494).

## Discussion

The purpose of the current study was to examine the associations among women's adverse experiences during childhood, their stressful life events during pregnancy, and four aspects of their social well-being: social support, couple aggression, neighborhood safety, and household food insecurity. There are three main findings. First, adverse experiences during childhood were associated uniquely with couple aggression and less perceived neighborhood safety, whereas stressful life events during pregnancy were associated uniquely with lower availability of social support and greater food insecurity. Second, adverse experiences during childhood and stressful life events were moderately correlated, resulting in an indirect pathway that linked adverse experiences during childhood with two aspects of women's social well-being through stressful life events during pregnancy: lower social support and food insecurity. Third, adverse experiences during childhood did not interact with recent stressful life events for any of the four examined aspects of women's social well-being.

The unique effects hypotheses associated with Aim 1 were partially supported given that only one of the two stressor/trauma variables was statistically significant in each analysis. Adverse experiences during childhood were uniquely associated with couple aggression for partnered women. This replicates the findings reported by Finy and Christian in their study of correlates of pregnant women's excessive inflammation (*i.e.*, C-reactive protein) and extends the unique effects of adverse childhood experiences to a relational aspect of women's everyday social lives.^31^

The finding also supports the contention forwarded by Thomas et al. regarding the importance of controlling for recent life events when examining the role of adversity during childhood in perpetuating the intergenerational transmission of women's stress and compromised functioning over the life course.^28^ This is important as it highlights a potential mechanism for the intergenerational transmission of health and well-being, such that mothers' adverse childhood experiences have lasting effects on biological and social functioning, both of which are important for women's and fetal health during pregnancy.

Childhood adverse experiences were also uniquely associated with women's perceptions of lower neighborhood safety. Although central to women's daily experiences, neighborhood conditions have rarely been examined in research on stressors and pregnant women's well-being. An exception is the study by Eick et al., which found that stressful life events were associated with lower perceived neighborhood quality.^17^ Their study, however, did not include a measure of adverse experiences during childhood. Given that stressful life events were not a unique correlate of perceived neighborhood safety in the current sample of women, our finding suggests that adverse experiences during childhood might serve as a unique, distal factor that shapes future limited housing and neighborhood options.

The long-term association between adverse childhood experiences and adults' perceived neighborhood safety seems to be a function, in part, of earlier contemporaneous interconnections during childhood and adolescence among neighborhood conditions and processes, family processes, socioeconomic status, and family members' well-being.^45–47^ This connection between childhood adversity and neighborhood options during adulthood invokes a structural life-course perspective that needs to be considered when examining pregnant women's health and well-being.^48^ The clinical and public health implications of the salience of neighborhood conditions bring to the forefront a need to consider the quality of women's near environments, in addition to physiological, personal, and relational aspects of their health and well-being during pregnancy.

The pattern of findings for pregnant women's social support and household food insecurity differed from those for couple aggression and neighborhood safety. Rather than unique associations for adverse childhood experiences, stressful life events during pregnancy were unique correlates of these aspects of women's social well-being, such that more recent stressful life events were associated with lower social supports. Our utilized measure of women's social support tapped into the availability and receipt of emotional, social, and instrumental support. The unique association between stressful life events and the receipt of social support found in the current study did not replicate findings reported by Lydsdottir et al.^49^ Using data provided by a large sample of pregnant women living in Iceland and controlling for a history of depression and adverse childhood experiences, they found that adverse events during adulthood were uniquely associated with concurrent mental health problems but not with social support.

Although other previous studies of life events and women's social support during pregnancy have not controlled for adverse experiences during childhood, research has suggested that social support is associated with life events during pregnancy either as a main effect or as a moderator offering protection against the deleterious effects of life events on women's mental health.^24,50,51^ Additional research is needed to discern the unique effects of stressful life events during pregnancy and women's social support.

Similar to women's social support, stressful life events during pregnancy were uniquely associated with food insecurity. This finding replicates the association found by Eick et al. in their large study of pregnant women living in San Francisco.^17^ They reported a correlation of 0.47 between life events and food insecurity, a magnitude similar to that found in the current study (*r* = 0.57). Their regression analyses also showed that food insecurity is related to but distinct from women's financial strain. The supplementary analyses we conducted also indicated that findings were more robust for the measure of food insecurity than the measure of income-to-needs. Taken together, the findings from these two studies suggest that food insecurity is an important aspect of pregnant women's socioeconomic well-being, and that future research and practice should distinguish among income, financial strain, and food insecurity.

The second main finding from the current study is the significant indirect pathway from adverse experiences during childhood to women's social support and household food insecurity *via* stressful life events during pregnancy. This indirect pathway is indicative of the environmental risk continuity proposition.^52^ The first component of this pathway is the association between adverse experiences during childhood (*i.e.*, neglect, abuse, household challenges) and stressful life events during pregnancy (*e.g.*, job loss, homelessness, legal trouble). Adjusting for covariates, the standardized association between these two stressor composites ranged from 0.41 to 0.46 (*p* < 0.001) across the four path analyses, suggesting that women who reported more adverse childhood experiences also reported more concurrent stressful life events.

The magnitude of this association was akin to a community sample of women living in Ohio (β = 0.43^31^) and less strong than that found in Icelandic women (β = 0.69^49^). The measures of adverse experiences during childhood and stressful life events during pregnancy are experience or event-based, rather than reports of perceived stress, and the two measures reflect events experienced at two different times across women's life courses.

The pathway from adverse experiences during childhood to pregnant women's well-being *via* stressful life events has been examined in previous studies with various well-being outcomes; the results have been mixed. In previous research, the indirect pathway was significant in relationship to women's mental health problems but not for perceived social support or pregnant women's inflammatory markers.^31,49^ Within the context of this sparse body of work, the significant results from the current study provide additional support for the proposition that adverse childhood experiences affect women's social well-being through stressful life events during pregnancy. A life-course perspective has been highlighted as useful for the consideration of pregnant women's lived experiences across their life course and their health.^53^ We suggest that the findings from the current study support the consideration of pregnant women's social well-being using a life-course perspective.

Future research should extend these findings by including additional explanatory mechanisms such as perceived stress, chronic cortisol production, and emotional dysregulation.^10,30,32^ There is accumulating evidence that adverse experiences during childhood provide an early-life explanation for the intergenerational transmission of risk and vulnerabilities across the life course.^54,55^ Future research is needed to ascertain if the selective nature of adverse experiences during childhood are a function of structural inequities such as discrimination in lending for housing and/or personal mechanisms such as epigenetic physiological stress responses, ineffective coping or emotional regulation, socioeconomic disadvantage, and challenging social and familial relationships.^28,31^ This future line of research will advance the understanding of the effects of adverse childhood experiences on pregnant women's well-being if both macro and micro features are considered simultaneously.

The third main finding is that adverse experiences during childhood did not interact with stressful life events during pregnancy in the analysis of women's social well-being. This interaction was tested to examine whether adverse childhood experiences amplified the deleterious effects of stressful life events on any of the four aspects of women's social well-being. Given that none of the interactions was statistically significant, there is little evidence of amplification effects from childhood adversity. To our knowledge, none of the studies that has examined this interaction previously has found significant effects.^28,49^ As such, current evidence suggests that adverse childhood experiences and stressful life events do not pile-up to reduce pregnant women's social well-being or increase their psychological health problems and compromised physiological stress responses.

### Limitations

This study enhances the understanding of how adverse childhood experiences and stressful life events during pregnancy are associated with women's social well-being, but there are important limitations. First, all the variables came from self-report questionnaires. This shared informant and method variance creates the potential to inflate the regression estimates.^56^ Although the nonsignificant statistical estimates in parts of the analytic models (*e.g.*, adverse childhood experiences not being uniquely associated with social support) suggest that the magnitude of spurious method effects in the current study are minimal, relying solely on questionnaire data remains an important limitation. Second, although the questionnaires to assess key variables focused on different time spans with some time ordering (*e.g.*, recalled childhood adversity, stressful experiences during pregnancy, current well-being), they were completed concurrently. Thus, women's current social well-being or recent stressful events could impact how they remember childhood experiences.^57^ This limitation could be addressed in future research with longitudinal data or with short-term designs that use intensive repeated measures.^58^

Third, the finding that none of the interactions terms between adverse childhood experiences and recent stressful life experiences were statistically significant might have resulted from low statistical power. The findings from the current study are consistent with the few other studies that have tested this interaction with various outcomes for pregnant women, but future studies that continue to examine this idea will profit from larger sample sizes.

Finally, although the sample was diverse with regard to socioeconomic status and racial-ethnic identification, the sample size was not large enough to conduct follow-up analyses that examined the extent to which the findings generalized to women with varying sociodemographic characteristics or circumstances. The generalizability of the findings will need to be considered in future research that has a larger, diverse sample of pregnant women.

## Conclusions

Adverse experiences during childhood and stressful life events during pregnancy place women and their newborns at risk for compromised health and well-being. In the current study, we substantiated that adverse and stressful experiences across the life course are associated with women's social well-being. Adverse childhood experiences were uniquely associated with perceptions and experiences that involved danger and overt negative interactions. Recent stressful life events were uniquely associated with reduced socioemotional, instrumental, and socioeconomic resources. As such, women's everyday lives during pregnancy are challenged when they do not perceive adequate social support, food security, or safe neighborhood conditions, as well as when they are in aggressive or violent intimate relationships.

We agree with recommendations that have been forwarded previously regarding the need to assess women's recent and historical stressful and traumatic experiences during routine prenatal care.^13,48,59^ This recommendation has important implications for medical professionals' training in trauma-related assessment and care. We extend these recommendations to include an assessment of multiple aspects of pregnant women's social well-being. Providing needed resources and supports during this pivotal time has the potential to interrupt the intergenerational transmission of physical, developmental, and psychosocial problems that stem, in part, from chronic and traumatic stressors.

## Data Availability

Data may be requested from the first author.

## Abbreviations Used

95% CI: 95% confidence interval
ACE: adverse childhood experience
CH: childhood
SD: standard deviation
SLE: stressful life events
